# Fluid intake survey among schoolchildren in Belgium

**DOI:** 10.1186/1471-2458-14-651

**Published:** 2014-06-26

**Authors:** Christelle Senterre, Michèle Dramaix, Isabelle Thiébaut

**Affiliations:** 1 Research Centre of Epidemiology, Biostatistics and Clinical Research, School of Public Health, Université Libre de Bruxelles, Route de Lennik, 808 Brussels, Belgium; 2 Club Européen des Diététiciens de l’Enfance, Brussels, Belgium

**Keywords:** Public health, Nutritional epidemiology, Nutritional education, Children nutrition, Fluid intake, Water intake, Beverages, Childhood obesity, Carbohydrate consumption, Added sugars

## Abstract

**Background:**

In childhood, inadequate fluid intakes can lead on the short term, to reduced physical and cognitive performances. However, few data are available on the fluid intake among schoolchildren in Belgium. The main aim of this study is to evaluate total fluid intake provided by different types of beverages in a sample of Belgian schoolchildren, in order to assess the percentage of individuals complying with the European Food Safety Authority recommendations for total fluid intake. A secondary aim was to characterize the study population in terms of determinants of the total fluid intake requirements.

**Methods:**

A child friendly “fluids and liquid food” diary was used to prospectively record the volume and frequency of beverage consumption over 7 days from 1045 schoolchildren. This diary also recorded the practice of physical activity. An adequate fluid intake was defined as an intake ≥ 75% of the age-specific adequate intake recommended by the EFSA.

**Results:**

The median (P25-P75) of habitual daily fluid intake was 864 (608–1104) ml/day, with 355 (194–579) coming from drinking water. This habitual daily fluid intake varied significantly among the three investigated EFSA groups (girls and boys aged from 8 years, girls from 9 to 13 and boys from 9 to 13), except for the drinking water (P = 0.906). The highest medians of fruit juice, sugar-sweetened beverages and milk and derivatives were found among boys of 9–13. Only 9.5% of the children had an adequate fluid intake, with a value of 19.2% among the 8 years old girls and boys, 7.0% among girls of 9–13 and 8.4% among boys of 9–13. In the whole sample, 27.7% of the children declared to drink less than 3-4x/day, 56% drunk water less than 2x/day and 7.7% drunk no water at all. Every day, 27.1% and 34.1% of the children drank respectively one fruit juice and one sugar-sweetened beverage.

**Conclusion:**

Belgian schoolchildren have an inadequate total fluid intake. Given the potential health consequences, interventions involving parents and school environment to promote water consumption seem pertinent.

## Background

Water is the major component of the human body and can therefore be considered as a construction element. However it also acts as a medium to support numerous metabolic reactions and to transport nutrients, hormones, waste products and heat in the body. Adequate hydration is consequently essential for life and for maintaining normal physical and cognitive performances [1]. An adequate hydration is obtained by balancing the sum of water intake, endogenous water production and the sum of water losses. Sources of water intake are fluids (drinking water and beverages), moisture content of foods and water produced by oxidative processes in the body. The amount of the metabolic water is however limited: it varies from about 250 to 350 ml/day in sedentary people to 600 ml/day in very active persons [2]. Requirements of water intake vary between individuals and according to environmental factors such as climate, physical activity and diet. Due to this variability, a minimum level of water intake cannot be used to set water requirements as the risk for water deficiency would be too high [1]. Therefore, the European Food Safety Authority (EFSA) defined adequate intakes (AI) for total water intake (water from fluids and food) from a combination of observed intakes in populations with desirable osmolarity values of urine and desirable water volumes per energy unit consumed [2]. For both boys and girls aged from 4 to 8 years, the adequate total water intakes are 1600 ml/day; for girls aged from 9 to13 years, they are 1900 ml/day and for boys of this age group, they are 2100 ml/day [2]. Since the predominant source of water intake are fluids (80%), and water from food contributes for only 20% of total water intake, the focus of this article will be on the fluid intake, and not total water intake [2]. The rational to assess the quantity of fluid intake can be found in recent research: a low fluid intake (<1 l/day) can lead on the short term, to a reduced physical and cognitive performances [3]. On the long term, a low water intake has been associated with the development of chronic kidney disease, lithiasis and hyperglycemia [4-6]. Even though these researches were performed among adults, similar research among children is merging [7,8]. Besides the quantity of fluid intake, the quality of fluid intake also needs to be assessed. According to the World Health Organization (WHO), a high consumption of sugar-sweetened beverages (SSBs) and juices could have a detrimental health effect, and an excessive consumption of SSBs is considered as a risk factor for overweight and obesity [9-12]. A cross sectional study (HELENA-CSS) collecting data of 2741 European adolescents (age 12.5-17.5 years) residing in 8 countries (Greece, Germany, Belgium, France, Hungary, Italy, Sweden, Austria and Spain) showed that sugar-sweetened beverages (SSBs) and juices represent an important part of the daily fluid consumption [13].

Even though prevalence of overweight and obesity among Belgian children aged 10 to 12 years was already remarkable in 2010 (respectively 13.2% boys and 11.2% girls, 3.7% boys and 2.3% girls), to the best of our knowledge, the consumption of SSBs, fruit juices and water among this age category has not yet been investigated [14].

Moreover, a recent survey among elementary school aged children in France suggested an inadequate water intake during the school day, warranting an assessment of fluid intake among Belgian schoolchildren [15]. Besides, after the survey, the school together with the parents could potentially educate or influence the fluid intake behavior of children, since they are an important reference for children [16,17].

The Belgian Consumption Food survey conducted in 2004 reported on fluid intake of Belgians older than 15 years and the results were reported on basic beverages for the fluid intake without energy. The mean daily consumption, evaluated on 24 hours was, for the 807 Belgians of 15 – 18 years, equal to 731 ml/day with 577 ml/day for the drinking water [18].

Given the limited available data on fluid intake among Belgian schoolchildren and the potential detrimental health effect of a low fluid intake and/or a high intake of SSBs, investigating the intake of drinking water and beverages of schoolchildren seemed pertinent. Therefore, the main aim of the present study was to evaluate total fluid intake provided by different types of beverages in a sample of children representative of the Belgian schoolchildren aged 8 to 13 years, in order to assess the percentage of individuals complying with the EFSA adequate intake for total fluid and for water intake. A secondary aim was to characterize the study population in terms of determinants of the total fluid intake requirements.

## Methods

### Study sample

The recruitment protocol of the children was based on the international protocol of the Health Behavior in School-aged Children study [19]. Based on a prevalence of 36% of adequate intake, observed in a French study, published in 2008, based on the beverage consumption in a sample of 1005 children aged from 6 to 11 years [20], a confidence interval of 95% and a precision of +/- 3%, the calculation of sample size, performed using Epi Info - version 3.5.3, suggested that 982 children should be included in the study. As the sampling protocol used has a design effect around 1.2 [19], the minimum number of subjects to include was equal to 1178 children. With the intention to have at least one class of each educational level (3^rd^ up to 6^th^) per school, and with the assumption that each class contained approximately 20 children, at least 80 children could be recruited per school and then 20 schools would be needed. Three random samples of 20 schools each were selected among the nearly 4500 elementary schools located in the 9 Provinces of Belgium: one main sample and two reserves. If the school principal accepted to participate in the study, all the pupils of the school from 3^rd^ to 6^th^ grade were eligible for the study. When a principal refused his consent, the school was replaced by a school, from the reserve samples, presenting, same characteristics. Finally, on one hand, 36 schools have refused to participate and, on the other hand, 13 schools have accepted the protocol and have participated to the study. Therefore, 1197 children of both genders, aged from 8 to 13 years, attending the second cycle of conventional primary school (3^rd^ up to 6^th^ grade) in Belgium were eligible for this study.

### Ethical aspects

The ethical committee of the Queen Fabiola Children’s University Hospital of Brussels approved (on 20 September 2011) the study protocol and all the documents distributed to the schools, children and parents (reference: CEH-N°58/11). An informed consent was signed by all participating children and their parents, and all the data were recorded anonymously.

### Data collection

Two visits took place in each school between February and June 2012. During the first visit, a letter of information addressed to the children and their parents, the informed consent and the nutritional questionnaire were handed over to the teachers. During the second visit, the completed questionnaires were collected and the child data (i.e. gender, date of birth and anthropometrical measurements) were recorded.

The “child” form recorded, for each subject, the anonyms study identification number, the gender, the date of birth and the anthropometrical measurements. These measurements were collected to characterize the study population in terms of determinants of water requirements. Children were weighted in standing position, in light clothing and stocking feet, to the nearest 0.1 kg, using a calibrate and transportable balance (Seca Elegantia 815®) accurate to 0.1 kg. Height (in cm) was measured, to the nearest 0.5 cm, with a portable microtoise (Seca 2131721009®). Waist circumference (in cm) was measured, at the narrowest point between the lower costal border and the iliac crest, at the end of expiration and above a T-shirt, with a tape measure (Nutricia®) to the nearest 0.1 cm.

A “fluids and liquid food” diary was used by the children to record prospectively their total liquid intake during 7 consecutive days [21,22]. The days of the diary were divided into 7 time slots: awakening and breakfast (6–10 AM), morning (10–12 PM), lunch (12–14 PM), afternoon (2–4 PM), afternoon snack (4–6 PM), dinner (6-8 PM), evening (8–10 PM) and night (10 PM-6 AM). Children were instructed to record, for each period, whether or not they drank anything. If they drank anything, details on the type and the volume of fluid, as well as details on the addition of sugar, fruit syrup, cacao or others, were requested. To help the children to report on the consumed volume, each child received a 250 milliliters (ml) graduated glass. If physical activity was performed, the type and the duration of the activity were also recorded.

To render the task of filling in the diary more entertaining and less dull, and consequently to reduce the rate of non-response, small smiley face stickers were used to fill out the diary. To avoid possible stigmatization, it was not the shape of the mouth of the smiley, but the color of the smiley face sticker that indicated the response. A green smiley sticker at a given location in the diary indicated the consumption of food or fluid item or the performance of a physical activity, whereas a blue smiley sticker indicated the opposite. Again to avoid stigmatization, the color red was avoided. For ensuring that the eagerly awaited tasks were clear and feasible, a pretest of the diary was done among 20 children.

### Variables

Based on the measures of weight and height, the BMI z-score of each child was calculated and the classification as normal weight, overweight or obesity was made in relation to the cutoffs set by the International Obesity Task Force (IOTF) [23]. Waist circumference, interpreted according to the child’s age, was used to define the presence of excessive body fat [24].

The mean daily physical activity frequency of each child was calculated as the total number of periods of physical activity reported, divided by the total number of days completed in the diary. The same calculation was made to estimate the mean daily duration of physical activity. WHO recommends, for children aged from 5 to 17 years, to accumulate at least 60 minutes of moderate- to vigorous-intensity physical activity daily and vigorous-intensity activities at least 3 times per week [25]. The frequency and the duration of habitual physical activity were categorized according to these recommendations into: “<1X/day” versus “≥1X/day”, “<60 min/day” versus “≥60 min/day” and “<3X/week” versus “≥ 3X/week”.

The “habitual daily fluid intake frequency” and the “habitual daily fluid intake volume” were calculated respectively as the total of fluid intakes and the total of the volume of fluids consumed over the period (independently of the type of fluid divided by the total number of days completed in the diary). A child was considered to have an adequate intake of fluids if his/her daily habitual fluid intake volume was ≥ 75% of the AI recommended by the EFSA for his/her age group. The EFSA assumes that 70 to 80% of the total water intake comes from fluids and 20 to 30% from food [2].

The types of fluid were classified as following: water, milk and derivatives, juices, SSBs, artificially sweetened beverages (diet beverages), energy drinks, alcoholic beverages and soup. The contribution of each fluid type, as a proportion of the mean daily total fluid volume was also calculated. The habitual daily consumption of sugar from fluids was estimated based on the habitual daily intake of fruit juices and SSBs. For this calculation, the sugar content of fruit juices is assumed to be 10% and those of SSBs 11% [26]. These percentages correspond to the minimum sugar content such fluid types could contain. The energy intake (kcal) from these fluid types was also calculated.

### Data processing and statistical analysis

On the 1197 eligible children, 34 have refused to participate. On the 1163 respondents; 1045 children have workable data. Data were entered using EpiInfo.6 and Excel 2010.

Proportions were used to present the BMI levels, the levels of physical activity and the adherence to EFSA adequate intake). Because total fluid intake, drinking water, fruit juice, SSB, milk and derivatives and sugar were skewness, median with both 25^th^ and 75^th^ percentiles were used to describe them.

Because the EFSA recommendations are formulated by age and gender, results were presented according to three groups: (1) the girls and the boys aged of 8 years, (2) the girls aged from 9 to 13 years and (3) the boys aged from 9 to 13 years. Because it is also well know that the intensity of physical activity had an influence on the fluid intake, the beverage consumption, and the adherence to the EFSA recommendations were presented according three levels of physical activity: (1) children who performed a physical activity ≥ 3×/week and for at least 60 min/day, (2) children with intermediate levels (i.e. ≥3×/week and < 60 min/day or < 3×/week and ≥60 min/day) and (3) children who performed a physical activity < 3×/week and less than 60 min/day.

All the comparisons of proportions according to the three EFSA groups or according to the three levels of physical activities were made with the help of the Pearson’s chi-square test (with the correction of Rao and Scott for take into account the design of the study). Asymmetric distributions were compared with the help of quantile regression with bootstrapped standard errors (for take into account the design of the study). The significance level for all tests was 0.05 and all statistical analyses were performed using Stata/SE 12.0 for Windows (TX: StataCorp LP).

## Results

The full analysis set contained the data of 1045 children and 91,7% of all children have completed all the 7 days of the fluid diary as requested. According to the EFSA groups, we have 16.7% of boys and girls aged to 8 years, 45.1% of girls aged from 9 to 13 years and 38.2% of boys aged from 9 to 13 years (Data not shown). Body mass index and physical activity levels are presented according to the three EFSA groups in Table 1. Independently of these three groups, overweight was diagnosed in 18.1% of the children, and obesity in 5.5% (Data not shown). These proportions were not statistically significantly different among the three EFSA groups (Table 1). Regarding the several variables related to physical activity, there were no statistically significantly variations among the three EFSA groups (Table 1). For all the children, the median (P25-P75) duration of physical activity was 32.1 (17.1-57.1) minutes per day (Data not shown). The median (P25-P75) of habitual daily fluid intake was 864 (608–1104) ml/day (Data not shown). The distributions of this habitual daily fluid intake were statistically significantly different (P = 0.036) according to the three EFSA groups, with the highest median observed among the 9–13 years old boys (920 ml/day vs. 836 ml/day for the 9–13 years old girls and 809 ml/day for the 8 years old boys and girls) (Table 2). For all the children, the several medians (P25-P75) of the different fluid types were: 355 (194–579) ml/day for the drinking water, 143 (71–240) ml/day for the juices, 175 (89–311) for the SSBs and 150 (79–250) for the milk and derivatives (Data not shown). Except the drinking water, which doesn’t varied statistically significantly (P = 0.906) among the three EFSA groups, the others fluid types were statistically significantly different among these groups, always with a higher median value among the 9–13 years old boys (Table 2). The median (P25-P75) amount of sugar provided by beverages investigated was equal to 38 (23–56) g/day (Data not shown). The quantity was statistically significantly higher among the 9–13 years old boys (43 g/day vs 36 g/day for the 9–13 years old girls and 35 g/day for the 8 years old boys and girls) (Table 2).

**Table 1:** BMI and physical activity levels, according to the three EFSA groups

	**Girls & boys,**	**Girls,**	**Boys,**	**P value**
	**8 years**	**9-13 years**	**9-13 years**	
BMI classes	(n = 162)	(n = 427)	(n = 361)	0.429
Normal	80.2	74.9	75.4	
Overweight	16.7	19.9	17.5	
Obese	3.1	5.2	7.1	
Physical activity	(n = 155)	(n = 419)	(n = 352)	0.052
≥ 1 x/day	16.1	9.6	23.3	
Physical activity	(n = 155)	(n = 419)	(n = 352)	0.118
≥ 60 min/day	24.8	17.5	29.8	
Physical activity	(n = 133)	(n = 372)	(n = 316)	
≥ 3 x/week	57.4	57.3	67.9	0.200

Data are %.

**Table 2:** Adequate intake, total fluid intake, drinking water, fruit juice, SSB, milk and derivatives and sugar, according to the three EFSA groups

	**Girls & boys,**	**Girls,**	**Boys,**	**P value**
	**8 years**	**9-13 years**	**9-13 years**	
	(n = 146)	(n = 416)	(n = 344)	
Adequate intake EFSA, %	19.2	7.0	8.4	<0.001
	(n = 146)	(n = 416)	(n = 344)	
Total fluid intake* (ml/day)	809 (513–1089)	836 (606–1061)	920 (671–1154)	0.036
	(n = 129)	(n = 395)	(n = 316)	
Drinking water (ml/day)	333 (179–554)	357 (207–571)	357 (165–614)	0.906
	(n = 117)	(n = 324)	(n = 260)	
Fruit Juice (ml/day)	121 (64–228)	136 (64–236)	163 (86–249)	0.003
	(n = 113)	(n = 351)	(n = 283)	
SSB (ml/day)	144 (86–279)	161 (82–268)	214 (100–369)	<0.001
	(n = 113)	(n = 331)	(n = 279)	
Milk & derivatives (ml/day)	143 (86–250)	143 (71–229)	171 (86–264)	0.046
	(n = 95)	(n = 280)	(n = 220)	
Sugar (g/day)	35 (22–57)	36 (22–51)	43 (25–62)	0.012

Data are % or Med (P25-P75).

*Total fluid intake is defined as water from drinking water and beverages and excludes water from food.

**Figure 1:**
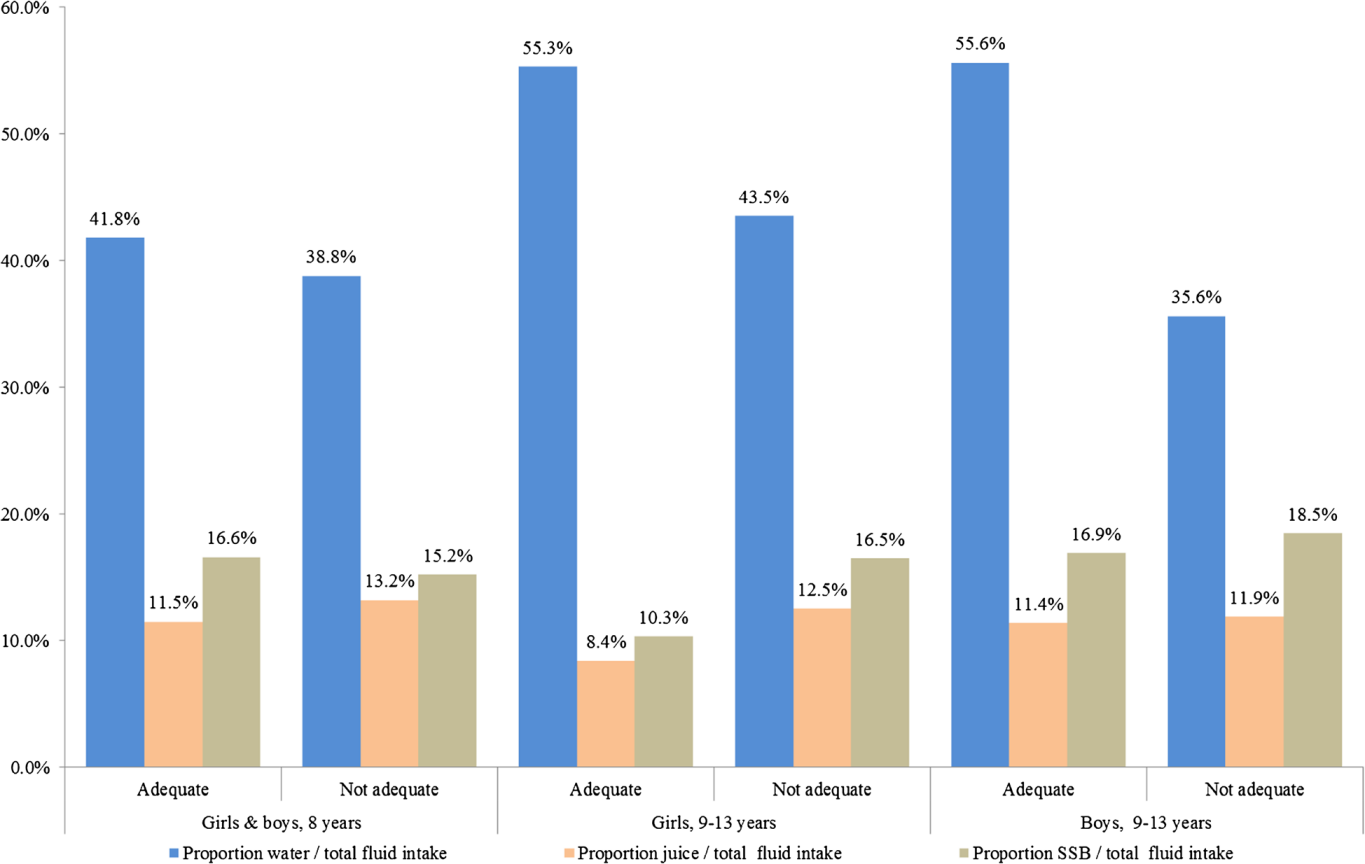
Proportion (%) of water, juice and SSBs intakes on the total fluid intake from beverages, according to the adequacy (or not) in the three EFSA groups.

The adequate intake for total fluid intake published by the EFSA was reached by 9.5% of all the children (Data not shown). The proportion of adequate intake differed statistically significantly among the three EFSA groups. The proportion of adequacy was the highest in the group of girls and boys aged to 8 years (19.2%) compared to the two others groups (7.0% for the 9–13 years old girls and 8.4% for the 9–13 years old boys) (Table 2). Based on the total fluid intake reported, the 8 years old girls and boys meet 67% of the recommended adequate intake and among the 9–13 years old, the proportions were nearly the same for the girls and the boys (58.7% and 58.2% respectively) (Table 3). On the whole, drinking water had contributed to 41.0% of the total fluid intake whereas juice and SSBs have contributed for 12.3% and 16.6% respectively (Data not shown). Regarding the variations of these contributions among the three EFSA groups, taking into account the fact to be or not in adequacy with the recommendations, we could see that the proportions of drinking water relative to the total fluid intake were always higher in the adequate group compared to the not adequate groups (Figure 1). On the other hand, the proportions of SSBs relative to the total fluid intake were, both for the girls and the boys aged from 9 to 13 years, higher for those who were not in adequacy with the recommendations (Figure 1). One in 4 children (27.7%) declared to drink less than 3-4×/day. When the number of fluid intakes was higher than 3-4×/day, the reported amount of water consumed tended to increase. More than half (56%) of the children declared to drink water less than 2×/day water and even 7.7% reported not to have drunk any water. Twenty-seven percent of the children drank one fruit juice every day and 34.1% drank one SSB every day. Milk and derivatives were not consumed daily by 71.8% of the children. Seventy-three % of the children reported to never consume soup (Data not shown).

**Table 3:** Median of the average total fluid intake per day (ml/day), and percentage of the adequate intake, according to the target groups of the EFSA recommendations

**EFSA groups**	Recommended* adequate intakes (AI)	**n**	Total fluid intake*** (ml/day) median (P25-P75)	**% of the AI**
Girls & boys,	1,600 ml/day	146	809 (513–1089)	67.4
≤ 8 years	with “70-80% provided by fluids”**			
	→ 1200 ml/day			
Girls,	1,900 ml/day	416	834 (606–1061)	58.7
9-13 years	with “70-80% provided by fluids”**			
	→ *1425 ml/day*			
Boys,	2,100 ml/day	344	920 (671–1154)	58.2
9-13 years	with “70-80% provided by fluids”**			
	→ *1575 ml/day*			

*EFSA recommendations, **we have used 75% which is the average of the 70-80% postulated.

***Total fluid intake is defined as water from drinking water and beverages and excludes water from food.

Practicing physical activity had a significant relationship on the consumption of total fluid intake and of drinking water. The medians of total fluid intake were statistically significantly (P < 0.001) different among the three levels of physical activity considered; with the highest median among the children who performed a physical activity ≥ 3×/week and for at least 60 min/day (Table 4). Statistically significant (P = 0.004) variation was also found regarding the drinking water consumption, with the lowest median observed among the children who performed a physical activity < 3×/week and less than 60 min/day (Table 4). Finally, the proportion of children who meet the recommendations of the EFSA was statistically significantly (P = 0.007) higher among the children who performed a physical activity ≥ 3×/week and for at least 60 min/day (15.8% vs. 10.3% for the intermediate level and 5.4% for the children who performed a physical activity < 3×/week and less than 60 min/day) (Table 4).

**Table 4:** Adequate intake, total fluid intake (defined as intake from drinking water and beverages, excluding water from foods), drinking water, % water/total fluid intake, fruit Juice, % juice/total fluid intake, SSB and % SSB/total fluid intake, according to the three physical activity levels

	**≥3x/week & ≥60 min/day**	Intermediate*	**<3x/week & <60 min/day**	**P value**
	(n = 184)	(n = 380)	(n = 242)	
Adequate intake EFSA, %	15.8	10.3	5.4	0.007
	(n = 202)	(n = 403)	(n = 257)	
Total fluid intake (ml/day)	995 (722–1265)	893 (664–1116)	818 (586–1018)	<0.001
	(n = 192)	(n = 383)	(n = 230)	
Drinking water (ml/day)	471 (254–717)	357 (196–586)	328 (167–496)	0.004
% water/total fluid intake	48.2	39.9	34.8	
	(n = 153)	(n = 320)	(n = 202)	
Fruit Juice (ml/day)	164 (71–257)	147 (75–236)	143 (64–236)	0.570
% juice/ total fluid intake	12.0	12.3	12.9	
	(n = 159)	(n = 341)	(n = 219)	
SSBs (ml/day)	196 (89–347)	181 (94–293)	157 (86–300)	0.192
% SSBs/total fluid intake	13.1	16.7	18.3	

*Intermediate is (≥3x/week & < 60 min/day) or (<3x/week & ≥60 min/day).

## Discussion

At the best of our knowledge, there are no other studies which have investigated the fluid consumption among school-aged children in Belgium. Our study is based on a large representative sample of whole the Belgium and not only based on a part of the country.

### Total fluid intake provided by beverages

As observed by other teams in others countries, the results of the survey showed an insufficient total fluid intake provided by beverages: The adequate intake for total fluid intake published by the EFSA was reached by only 9.5% of all the children. A recent survey among French schoolchildren aged from 9 to 11 years showed that 2/3 of the children had a low fluid intake in the morning after breakfast; and at breakfast 73.5% of students drank less than 400 ml [15]. Four other European studies showed the following results: the average volume consumed by 593 French children aged from 3 to 9 years was 838 ml/day, an Italian study reported a volume of 744 ml consumed by 138 children aged from 1 to 9 years and the German study among 363 children aged from 4 to 10 years describe an average fluid intake ranging from 520 to 690 ml/day [27-29]. In the “Donald” cohort study, the observed values were very similar to those found in the present study: the mean fluid intake for girls and boys aged 9–13 years was 823 ml/day and 969 ml/day respectively [30].

### Drinking water

Our results on water intake (333 ml/day for children aged from 8 years, 357 ml/day for the girls aged from 9 to 13 years and 357 ml/day for the boys of the same age group) are also comparable to those observed among the German children: 215 ml/day for children aged from 4 to 8 years, 344 ml/day for boys from 9 to 13 years and 298 ml/day for girls of the same age [30]. The 8-year-old children of our sample drank on average 333 ml/day. This seems higher than in children of the German cohort, but this difference is probably due to the younger age of the German subpopulation. Among American children and adolescents aged from 2 to 18 years, consumption data recorded in 2005–2006 revealed a mean water intake of 552 ml/day. Thirty years ago (1977–1978) the mean water consumption among this population was 624 ml/day [31]. A Brazilian study among children and adolescents reported a mean water consumption of 480 ml/day among the 3–6 years old, 610 ml/day among the 7–10 years old and 700 ml/day among the 11–17 years old [32]. The difference in climate between Belgium and Brazil could be a possible explanation for the difference in water intake. However, the contribution of water to the total fluid intake in this Brazilian study represented only 33-34% whereas in the present study the contribution of water was 41%.

Given the low water intake observed among children, a concern about the potential impact on their hydration status and their health is legitimate. A low water intake, less than 50% of the total fluid, will be compensated by the intake of other beverages that will indeed contribute to the maintenance of the hydration balance but also to a positive energy balance. Moreover, a low water intake has been shown to affect the cognitive abilities of children. Recent studies have shown a link between hydration status and cognitive performance and the appearance of headache [7,8,31,33,34]. A striking, yet positive observation made from the current results is that by increasing the daily consumption of water, and not SSBs, the AI for total water intake was achieved.

Performing a physical activity causes a water loss and demands adequate hydration [1,31]. Children in this study who practice a physical activity at least 3 times per week and for a minimum duration of 60 minutes per day consumed significantly more water (471 ml/day) than the less physically active children (357 ml/day for the intermediate level of physical activity and 328 ml/day for the children who practice a physical activity less than 3 times per week and less than 60 minutes per day). More extensive and targeted research is necessary to determine whether this higher water intake is sufficient to compensate for the water loss induced by the physical activity.

### Fruit juices and SSBs

The most consumed fluids after water were SSBs and fruit juices. They represent, respectively, on average, 16.6% and 12.3% of the habitual total fluid intake. The habitual median (P25-P75) intake of SSBs per child was 175 (89–311) ml/day and of fruit juice 143 (71–240) ml/day.

Compared to the present study, the “Donald” cohort study reported a very similar level of consumption of fruit juice and of sugary drinks [30]. The children in the present study consumed more sugary drinks than fruit juice while the German schoolchildren aged from 4 to 9 years consumed more fruit juice than sugary drinks [30]. In a review on hydration and fluid intake behaviors among children, Bresson and Goudable [35] referred to the results of several epidemiological studies showing an inadequate total water intake due to a low water intake on one hand and a high intake of SSBs and fruit juices on the other hand.

According to the National Health Council, the energy need of a child, aged from 8 to 12 years with a light physical activity level, ranges from 53 to 60 kcal/kg/day for boys and from 47 to 57 kcal/kg/day for girls. The intake of added sugars should not exceed 10% of total energy intake [36]. Applying this recommendation to the children in the present study, the energy requirement is 2094 kcal/day (56 kcal*37.4 kg) with a maximum of 209 kcal from added sugars for the boys and 1924 kcal (52 kcal*37 kg) with a maximum of 192 kcal from added sugars for the girls. The observed mean energy intake from sugar derived from beverages is 169 kcal/day and 140 kcal/day for boys and girls respectively. This energy input is 3/4 of the recommended upper limit of added sugars by the National Health Council. Since added sugars from beverages are now recognized as a risk factor for excessive weight gain [12,37-40], this energy input is alarming because the children of the present study already have a mean weight above the 90^th^ percentile on the international curves; one fourth of them is overweight or obese and 70% of them do not reach 60 minutes/day of physical activity. This cross-sectional study naturally does not imply a causal link between overweight or obesity and sugar consumption. However as mentioned by the WHO, these findings of a high consumption of soft drinks and juices are likely to lead an entire young population to an increased risk for non-communicable diseases such as obesity and dental caries disease [9,10].

### Soup

Soup is apparently no longer part of the eating habits of the young generation as 73.1% of the selected children never eat soup. Among the consumers of soup, the habitual amount was 64.3 ml/day. This portion size is not even equivalent to one of the five recommended daily portions of fruit and vegetables.

### Milk and dairy drinks

The milk is under-consumed by the children in the study: 71.8% of children do not consume milk or milk drinks daily and, among the consumers, the habitual amount was equal to 150.0 ml/day. The “Donald” cohort study showed that the range of milk consumption among 4 to 13 year old children was 144 to 203 ml/day [30]. This study also highlighted a declining trend in milk consumption. The same trend was observed in the NHANES survey: the number of young children who drink milk decreased significantly over the last decade while the consumption of fruit juice dramatically increased [41]. They also pointed out that milk was the main source of calcium for children. Although our study population is older, the same alarming conclusion about calcium intake derived from milk and milk drinks was drawn.

### Frequency of consumption

The survey method and the «fluids and liquid food» diary made it possible to objectively determine the frequency of consumption of each fluid type. As expected, all children consumed at least one drink per meal, 3 to 4 times/day (breakfast, lunch, snack and dinner).

The low frequency of water consumption raised questions about the availability of water and its accessibility at home, but also at school where children spend most of the day or during outdoor activities. Various studies investigated the accessibility to water in schools and the impact on fluid intake [18,42]. Teachers have recognized an improvement in concentration and learning of their pupils when they had free access to water [8,34]. Bresson and Gaudable [35] suggested that a possible option to promote water consumption includes educational programs involving different people present in the lives of children like parents, teachers and medical and social workers. They proposed to enhance water consumption by providing information on the health benefits of water and by increasing the accessibility to water in the daily environment of the child, including school.

### Strengths and limits of the study

The fluid intake data presented in this article were obtained by a fluid diary completed over 7 consecutive days by the children with the help of their teachers and parents. The volume of fluid consumed was objectively measured by the use of a graduated cup.

The use of a specific recording instrument suitable for children seems to be the most appropriate method for assessing the amount of liquids consumed and the amount of water volumes [43,44]. Conventional methods of food records (food diaries) covering all the different meals over several days, were criticized because these instruments risk to not accurately record the volume and the type of drinks consumed outside meals possibly due to forgetfulness [43,44]. In the present study, the children completed each day their specific fluid diary and this over one week. On the one hand, this method is time demanding and expensive, while, on the other hand, the recall bias is minimized as it is completed at the time of consumption. However, in order to complete this diary, children, need to be able to read and write, which could result in a selection bias. Yet, the selection bias in this study was even reduced by the fact that the completion of the fluid diary was integrated in the school activities. This also could explain the good participation rate of 87% to the recording of the diary. A participation rate of 30% is generally accepted as a good recruitment. In addition, the completion rate for the entire sample was 91.7%, which means that 916 children out of the 999 children have properly completed the full 7 days of the diary.

A potential overestimation of the total fluid intake should not be disregarded. The child could possibly take the smiley stickers used to complete the diary as a game and consequently they could have been stimulated to drink. The study protocol was however designed in order to avoid all other possible stimuli from persons outside the usual environment of the child to complete the diary. Looking at the results, an overestimation seems unlikely because the observed volumes are close to those observed in other studies.

## Conclusion

The main objective of the study was to estimate the volume of water and other beverages consumed by Belgian schoolchildren aged 8 to 12 years and to compare these intakes to the current EFSA recommendations. The results showed a low total fluid intake provided by beverages (<1 liter/day) with a contribution from water (41.3%) too small compared to the contribution from fruit juice and sweetened beverages (12.3% and 16.6% respectively). Twenty five percent of the children drank less than 3 to 4 times per day. Another observation was the low consumption of “healthy liquid food” by these schoolchildren. The girls had a healthier behavior as they chose more frequently water than boys who preferred sugary drinks. The most physically active children consumed daily more water than the less physically active children. If children want to reach the adequate intake on total water, a minimum contribution of 55% of water to the total fluid intake seems essential, with a consumption distributed on at least 3 to 4 times per day.

## Abbreviations

AI: Adequate Intake; BMI: Body Mass Index; EFSA: European Food Safety Authority; IOTF: International Obesity Task Force; SSBs: Sugar-Sweetened Beverages; WHO: World Health Organization.

## Competing interests

This study was funded by Danone waters Benelux, but it had no role in study design, data collection and analysis, and preparation of the manuscript. The authors declare that they have no other competing interests.

## Authors’ contribution

All authors were involved in the conception of the study, the study design, the supervision of data collection, the statistical analyses, the interpretation of the data and the drafting of the manuscript. All authors read and gave final approval for the version submitted for publication.

## Pre-publication history

The pre-publication history for this paper can be accessed here:

http://www.biomedcentral.com/1471-2458/14/651/prepub

## References

[B1] Jéquier E Constant F Water as an essential nutrient: the physiological basis of hydration Eur J Clin Nutr 2010 64 2 115 123 19724292 10.1038/ejcn.2009.111

[B2] European Food Safety Authority Scientific opinion on dietary reference values for water EFSA J 2010 8 3 14595

[B3] Pross N Demazieres A Girard N Barnouin R Metzger D Klein A Perrier E Guelinckx I Effects of changes in water intake on mood of high and low drinkers PLoS One 2014 9 4 e94754 doi: 10.1371/journal.pone.0094754 24728141 10.1371/journal.pone.0094754 PMC3984246

[B4] Strippoli GF Craig JC Rochtchina E Flood VM Wang JJ Mitchell P Fluid and nutrient intake and risk of chronic kidney disease Nephrology (Carlton) 2011 16 326 334 21342326 10.1111/j.1440-1797.2010.01415.x

[B5] Curhan GC Willett WC Knight EL Stampfer MJ Dietary factors and the risk of incident kidney stones in younger women: Nurses’ Health Study II Arch Intern Med 2004 164 885 891 15111375 10.1001/archinte.164.8.885

[B6] Roussel R Fezeu L Bouby N Balkau B Lantieri O Alhenc-Gelas F Marre M Bankir L D.E.S.I.R Study Group Low water intake and risk for new-onset hyperglycemia Diabetes Care 2011 34 2551 2554 21994426 10.2337/dc11-0652 PMC3220834

[B7] Edmonds CJ Jeffes B Does having a drink help you think? 6-7-Year-old children show improvements in cognitive performance from baseline to test after having a drink of water Appetite 2009 53 3 469 472 19835921 10.1016/j.appet.2009.10.002

[B8] D'Anci KE Constant F Rosenberg IH Hydration and cognitive function in children Nutr Rev 2006 64 10 457 464 17063927 10.1301/nr.2006.oct.457-464

[B9] National Health and Nutrition Examination Survey[http://www.cdc.gov/nchs/nhanes.htm]

[B10] Organisation Mondiale de la Santé Rapport sur la santé bucco-dentaire dans le monde 2003. Poursuivre l’amélioration de la santé bucco-dentaire au XXIe siècle – l’approche du Programme OMS de santé bucco-dentaire 2003 Genève: L’Organisation Mondiale de la Santé

[B11] Ernährungskommission der Deutschen Gesellschaft für Kinder- und Jugendmedizin, Ernährungskommission der Österreichischen Gesellschaft für Kinder- und Jugendmedizin, Commission de nutrition de la Société Suisse de Pédiatrie Consommation de boissons sucrées par les enfants et les adolescents Paediatrica 2008 19 4 29 30

[B12] Collison KS Zaidi MZ Subhani SN Al-Rubeaan K Shoukri M Al-Mohanna FA Sugar-sweetened carbonated beverage consumption correlates with BMI, waist circumference, and poor dietary choices in school children BMC Public Health 2010 10 234 20459689 10.1186/1471-2458-10-234 PMC2877673

[B13] Duffey KJ Huybrechts I Mouratidou T Libuda L Kersting M DeVriendt T Gottrand F Widhalm K Dallongeville J Hallstrôm L Gonzalez-Gross M Dehenauw S Moreno LA Popkin BM Beverage consumption among European adolescents in the HELENA Study Eur J Clin Nutr 2012 66 2 244 252 21952695 10.1038/ejcn.2011.166 PMC3392586

[B14] International Obesity Taskforce [http://www.worldobesity.org]

[B15] Bonnet F Lepicard E Cathrin L Letellier C Constant F Hawili N Friedlander G French children start their school day with a hydratation deficit, France Ann Nutr Metab 2012 60 257 263 22677981 10.1159/000337939

[B16] Molloy CJ Gandy J Cunningham C Slattery G An exploration of factors that influence the regular consumption of water by Irish primary school children J Hum Nutr Diet 2008 21 5 512 515 18833589 10.1111/j.1365-277x.2008.00880.x

[B17] Kaushik A Mullee MA Bryant TN Hill CM A study of the association between children's access to drinking water in primary schools and their fluid intake: can water be ‘cool’ in school? Child Care Health Dev 2007 33 4 409 415 17584396 10.1111/j.1365-2214.2006.00721.x

[B18] Debacker N Temme L Cox L Huybrechts I Van Oyen H Institut Scientifique de Santé Publique Consommation d’aliments Enquêtes de consommation alimentaire belge 2004 2007 19 43

[B19] Health Behaviour in School-aged Children. World Health Organization Collaborative Cross-National Survey[http://www.hbsc.org]

[B20] Centre de Recherche pour l’Etude et l’Observation des Conditions de Vie[http://www.credoc.fr]

[B21] Lafay L Mennen L Six MA Calamassi-Tran G Hercberg S Volatier JL Castetbon K Ambroise Martin A Etude de validation d’un carnet de consommation alimentaire de 7 jours pour INCA2-ENNS. Insee-Méthodes: Actes des Journées de Méthodologie Statistique 2002 Paris: L’Insee

[B22] Agence Française de Sécurité Sanitaire des Aliments Etude Individuelle Nationale des Consommations Alimentaires 2 (Inca 2) 2006–2007 2009 Paris: L’Agence nationale de sécurité sanitaire de l’alimentation, de l’environnement et du travail

[B23] Cole T Bellizzi M Flegal K Dietz W Establishing a standard definition for child overweight and obesity worldwide: international survey BMJ 2000 320 1 6 10797032 10.1136/bmj.320.7244.1240 PMC27365

[B24] Taylor RW Jones IE Williams SM Goulding A Evaluation of waist circumference, waist-to-hip ratio, and the conicity index as screening tools for high trunk fat mass, as measured by dual-energy X-ray absorptiometry, in children aged 3–19 y, USA Am J Clin Nutr 2000 72 490 495 10919946 10.1093/ajcn/72.2.490

[B25] Organisation Mondiale de la Santé Recommandations mondiales sur l’activité physique pour la santé[http://whqlibdoc.who.int/publications/2010/9789242599978_fre.pdf]

[B26] Nubel Food Planner 2010 5 [http://www.nubel.be]

[B27] Volatier J-L Enquête Inca - Enquête Individuelle et Nationale sur les Consommations Alimentaires. Collection AFSSA 2000 Paris: Tec & Doc Lavoisier

[B28] Turrini A Saba A Perrone D Cialfa E D’Amicis A Food consumption patterns in Italy: the INN-CA Study 1994–1996 Eur J Clin Nutr 2001 55 571 588 11464231 10.1038/sj.ejcn.1601185

[B29] Manz F Wentz A Hydration status in the United States and Germany Nutr Rev 2005 63 6 Pt 2 S55 S62 16028572 10.1111/j.1753-4887.2005.tb00154.x

[B30] Sichert-Hellert W Kersting M Manz F Fifteen year trends in water intake in German children and adolescents: results of the DONALD study: Dortmund Nutritional and Anthropometric Longitudinally Designed Study Acta Paediatr 2001 90 7 732 737 11519974

[B31] Popkin B D’Anci K Rosenberg I Water, hydratation and health USA Nutr Rev 2010 68 8 439 458 10.1111/j.1753-4887.2010.00304.x PMC2908954 20646222

[B32] Feferbaum R de Abreu LC Leone C Fluid intake patterns: an epidemiological study among children and adolescents in Brazil BMC Public Health 2012 12 1005 23167254 10.1186/1471-2458-12-1005 PMC3507861

[B33] Benton D Burgess N The effect of the consumption of water on the memory and attention of children Appetite 2009 53 1 143 146 19445987 10.1016/j.appet.2009.05.006

[B34] Fadda R Rapinett G Grathwohl D Parisi M Fanari R Calò CM Schmitt J Effects of drinking supplementary water at school on cognitive performance in children Appetite 2012 59 3 730 737 22841529 10.1016/j.appet.2012.07.005

[B35] Bresson JL Goudable J Hydratation de l’enfant et comportement dipsique Cah Nutr Diét 2013 48 41 52

[B36] Conseil Supérieur de la Santé Recommandations nutritionnelles pour la Belgique (révision 200) Publication du CSS n°8309 2009 Bruxelles: Le Conseil Supérieur de la Santé

[B37] De Ruyter J Olthof M Seidell J Katan M A trial of sugar-free or sugar-sweetened beverages and body weight in children N Engl J Med 2012 367 1397 1406 22998340 10.1056/NEJMoa1203034

[B38] Qi Q Chu AY Kang JH Jensen MK Curhan GC Pasquale LR Ridker PM Hunter DJ Willett WC Rimm EB Chasman DI Hu FB Qi L Sugar-sweetened beverages and genetic risk of obesity N Engl J Med 2012 367 1387 1396 22998338 10.1056/NEJMoa1203039 PMC3518794

[B39] Ebbeling CB Feldman HA Chomitz VR Antonelli TA Gortmaker SL Osganian SK Ludwig DS A randomized trial of sugar-sweetened beverages and adolescent body weight N Engl J Med 2012 367 1407 1416 22998339 10.1056/NEJMoa1203388 PMC3494993

[B40] Bergouignan A Blanc S Simon C “Calories” et obésité: quantité ou qualité? Cah Nutr Diét 2010 45 180 189

[B41] Fulgoni VL 3rd Quann EE National trends in beverage consumption in children from birth to 5 years: analysis of NHANES across three decades Nutr J 2012 11 92 23113956 10.1186/1475-2891-11-92 PMC3551691

[B42] Muckelbauer R Libuda L Clausen K Toschke AM Reinehr T Kersting M Promotion and provision of drinking water in schools for overweight prevention: randomized, controlled cluster trial Pediatrics 2009 123 4 661 667 10.1542/peds.2008-2186 19336356

[B43] Vergne S Methodological aspects of fluid intake records and surveys Nutrition Today 2012 47 7 10

[B44] Thompson F Subar A Coulston A, Rock C, Monsen Dietary assessment methodology Nutrition in the Prevention and Treatment of Disease 2001 San Diego: Academic Press 3 22

